# Deciphering *Aspergillus* section *Terrei* in *Galleria mellonella* model: a clade-specific pathogenicity characterization

**DOI:** 10.1128/spectrum.02576-24

**Published:** 2025-03-17

**Authors:** Roya Vahedi-Shahandashti, Jos Houbraken, Vit Hubka, Martin Meijer, Bettina Gudrun Zelger, Ulrike Binder, Cornelia Lass-Flörl

**Affiliations:** 1 Institute of Hygiene and Medical Microbiology, Medical University of Innsbruck, ECMM Excellent Center of Mycology, ISHAM Working Group Member of *A. terreus*, Innsbruck, Austria; 2 Westerdijk Fungal Biodiversity Institute 141042 , Utrecht, the Netherlands; 3 Department of Botany, Faculty of Science, Charles University 580016 , Prague, Czech Republic; 4 Laboratory of Fungal Genetics and Metabolism, Institute of Microbiology, Czech Academy of Sciences 86863 , Prague, Czech Republic; 5 Institute of Pathology, Neuropathology and Molecular Pathology, Medical University of Innsbruck 27280 , Innsbruck, Tyrol, Austria; Universidade de Sao Paulo, Ribeirao Preto, Sao Paulo, Brazil

**Keywords:** *Aspergillus terreus*, antifungal susceptibility testing, antifungal resistance, pathogenicity, histopathology, virulence, section *Terrei*, *Galleria mellonella* model

## Abstract

The *Aspergillus* genus encompasses a diverse array of species, some of which are opportunistic pathogens. Traditionally, human aspergillosis has primarily been linked to a few *Aspergillus* species, predominantly *A. fumigatus*. Changes in epidemiology and advancements in molecular techniques have brought attention to less common and previously unrecognized pathogenic cryptic species. Despite the taxonomic recognition of many cryptic species in section *Terrei*, their virulence potential and clinical implications, compared to *A. terreus sensu stricto*, remain poorly understood. Hence, the current study utilized the alternative *in vivo* model *Galleria mellonella* to evaluate the virulence potential of 19 accepted *Aspergillus* species in section *Terrei*, classified into three series (major phylogenetic clades): *Terrei*, *Nivei*, and *Ambigui*. Analyzing the median survival rates of infected larvae of all species in each series revealed that series *Ambigui* has a significantly lower virulence compared to series *Terrei* and *Nivei*. Taking a closer look at series *Terrei* and *Nivei* revealed a trend of survival within each clade, dividing the species into two groups: highly virulent (up to 72 h survival) and less virulent (up to 144 h survival). Histological observation, considering fungal distribution and filamentation, further supported this assessment, revealing increased distribution and hyphal formation in virulent species. Additionally, the susceptibility profile of conventional antifungals was determined, revealing an increased azole minimum inhibitory concentration for some tested cryptic species such as *A. niveus* and *A. iranicus*. Our results highlight the importance of cryptic species identification, as they can exhibit different levels of virulence and show reduced antifungal susceptibility.

**IMPORTANCE:**

With changing fungal epidemiology and an increasingly vulnerable population, cryptic *Aspergillus* species are emerging as human pathogens. Their diversity and clinical relevance remain underexplored, with some species showing reduced antifungal susceptibility and higher virulence, highlighting the need for better preparedness in clinical practice. Using the *Galleria mellonella* model, we assessed the virulence of *Aspergillus* species of section *Terrei*, including cryptic and non-cryptic species, across three series *Terrei*, *Nivei*, and *Ambigui*. The results revealed significant virulence variation among the series, with some cryptic species displaying high virulence. Histological analysis confirmed increased hyphal formation and fungal spread in the more virulent species. Additionally, elevated azole minimum inhibitory concentrations were also observed in certain cryptic species. This study presents novel insights into the pathogenicity of *Aspergillus* section *Terrei*, emphasizing the critical importance of accurately identifying cryptic species due to their diverse virulence potential and antifungal resistance, which may have substantial clinical implications.

## INTRODUCTION

Invasive aspergillosis (IA) stands out as one of the most severe clinical fungal infections primarily affecting immunocompromised patients (1). It is characterized by significant morbidity and mortality, reaching up to 50% in certain groups of patients, highlighting the substantial impact of this infection on healthcare systems (1–3). As saprophytic fungi, *Aspergillus* species can adapt to challenging environments, assisting the fungus in resisting human host defenses and playing a significant role in causing a range of complicated infections (3). While *A. fumigatus* is the most common species isolated from patients with IA, other *Aspergillus* species, such as *A. terreus sensu stricto* (s.s.), can also be causative agents (4, 5). Managing infections caused by *A. terreus* is challenging due to its reduced sensitivity to amphotericin B (AMB), resulting in a lower response rate to the limited available options of antifungal therapy (6–8). There are currently 25 accepted *Aspergillus* species in section *Terrei*, grouped into three series (major phylogenetic clades): *Terrei*, *Nivei*, and *Ambigui* (9). *Aspergillus terreus* s.s. has been reported as the most frequently isolated clinical species in section *Terrei*, followed by *A. citrinoterreus* (10). Many *Aspergillus* species in section *Terrei* are commonly referred to as cryptic species, and they are often underdiagnosed compared to their non-cryptic counterparts (11). This underdiagnosis arises from several factors, including inaccurate species identification in databases, incomplete matrix-assisted laser desorption/ionization-time of flight (MALDI-TOF) libraries, and the reliance of most clinical microbiology laboratories on morphology-based identification methods, which cannot differentiate cryptic species that are defined solely by molecular data and phylogeny (12, 13).

Beyond the taxonomic perspective, cryptic species are gaining clinical attention (11). The presence of cryptic *Aspergillus* species in clinical samples is increasingly reported with the introduction of sequence-based identification, thereby altering the epidemiology of aspergillosis (14, 15). Cryptic species are highly intriguing, as different species exhibit varying susceptibilities to multiple antifungal drugs, thus influencing the selection of appropriate antifungal therapy (13, 16, 17). Infections associated with cryptic species are less documented from both clinical and epidemiological viewpoints, primarily due to challenges in routinely achieving accurate identification universally (18). Given the dynamic epidemiology of fungal infections and the identification of cryptic species in clinical specimens, along with their diminished susceptibility to antifungal agents, their clinical relevance is underscored. The present study aimed to characterize the virulence of 19 out of 25 currently accepted species within section *Terrei*, as listed in the curated species list (9), via survival analysis and histological examination in *Galleria mellonella* larvae, and determine their *in vitro* antifungal susceptibility patterns.

## RESULTS

### Phylogenetic insight into antifungal susceptibility patterns

**Fig 1:**
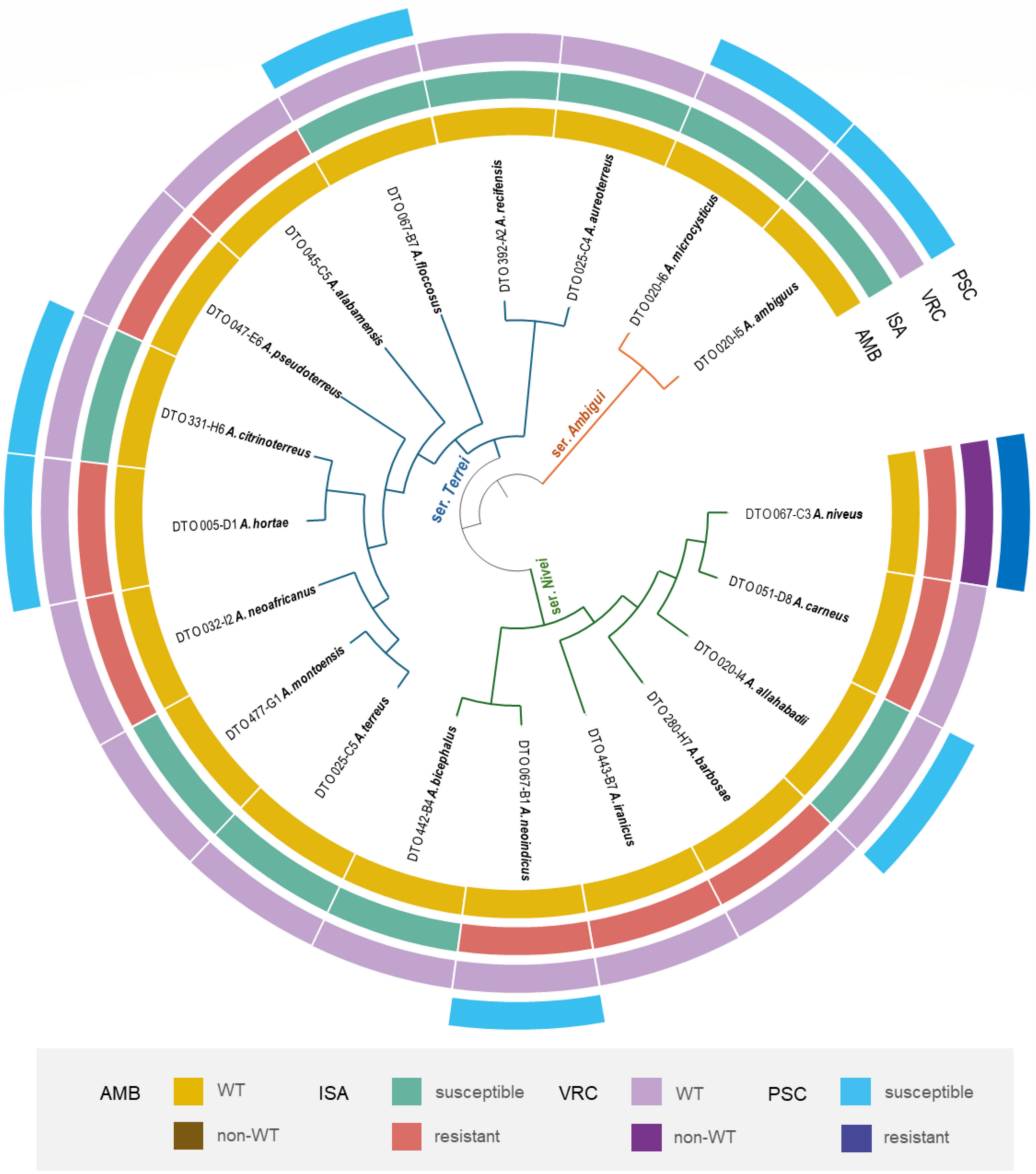
Phylogenetic overview of *Aspergillus* section *Terrei* with division into series. Antifungal susceptibility based on clinical breakpoints or epidemiological cut-off value (ECOFF) outlined by EUCAST 2022 protocol E.DEF 9.4 is plotted on the tree. AmB, amphotericin B; ISA, isavuconazole; Non-WT, non-wild type; PSC, posaconazole; VRC, voriconazole; WT, wild type.

The species included in this study, each represented by one isolate, and their susceptibility profiles to conventional antifungals are detailed in Table 1. The phylogenetic relationships between tested species are shown in Fig. 1. Nineteen tested species clustered into three main clades corresponding to series *Terrei*, *Nivei*, and *Ambigui* (Fig. 1).

**TABLE 1:** Antifungal susceptibility profile of *Aspergillus* section *Terrei* strains utilized in this study^*a*^

Series	Species	Isolate number	MIC/MEC (mg/L)					
			AmB	VRC	PSC	ISA	CAS	MCF
*Terrei*	*Aspergillus terreus s.s*.	DTO 025-C5 (CBS 601.65)	2	1	0.25	0.5	0.5	0.03
	*Aspergillus citrinoterreus*	DTO 331-H6 (CBS 138921)	4	1	0.125	1	0.5	0.03
	*Aspergillus aureoterreus*	DTO 025-C4 (CBS 503.65)	1	1	0.125	1	0.25	0.03
	*Aspergillus pseudoterreus*	DTO 047-E6 (CBS 123890)	1	2	0.25	2	0.5	0.03
	*Aspergillus recifensis*	DTO 392-A2	4	1	0.125	0.5	0.125	0.03
	*Aspergillus floccosus*	DTO 067-B7 (CBS 116.37)	4	1	0.125	1	0.125	0.06
	*Aspergillus neoafricanus*	DTO 032-I2 (CBS 125688)	1	1	0.25	2	0.25	0.03
	*Aspergillus alabamensis*	DTO 045-C5 (CBS 125693)	1	2	0.25	2	0.5	0.03
	*Aspergillus hortae*	DTO 005-D1 (CBS 811.96)	1	0.5	0.06	2	0.5	0.03
	*Aspergillus montoensis*	DTO 477-G1 (CBS 149107)	2	0.5	0.25	1	0.25	0.03
*Nivei*	*Aspergillus niveus*	DTO 067-C3 (CBS 115.27)	2	4	0.5	4	0.25	0.06
	*Aspergillus carneus*	DTO 051-D8 (CBS 494.65)	1	1	0.25	2	0.25	0.03
	*Aspergillus neoindicus*	DTO 067-B1 (CBS 444.75)	1	2	0.125	2	0.25	0.03
	*Aspergillus iranicus*	DTO 443-B7 (CBS 139561)	2	1	0.25	4	0.125	0.03
	*Aspergillus allahabadii*	DTO 020-I4 (CBS 164.63)	4	1	0.125	1	0.25	0.03
	*Aspergillus barbosae*	DTO 280-H7	1	0.5	0.25	2	0.25	0.03
	*Aspergillus bicephalus*	DTO 442-B4 (CBS 142900)	2	2	0.25	1	0.25	0.03
*Ambigui*	*Aspergillus microcysticus*	DTO 020-I6 (CBS 120.58)	0.5	0.5	0.06	0.25	0.125	0.03
	*Aspergillus ambiguus*	DTO 020-I5 (CBS 117.58)	1	1	0.06	1	0.125	0.03
	MIC range	*Terrei*	1–4	0.5–2	0.06–0.25	0.5–2	0.125–0.5	0.03–0.06
		*Nivei*	1–4	0.5–4	0.125–0.5	1–4	0.125–0.25	0.03–0.06
		*Ambigui*	0.5–1	0.5–1	0.06	0.25–1	0.125	0.03

^*a*^ MIC, minimum inhibitory concentration; MEC, minimum effective concentration; AmB, amphotericin B; VRC, voriconazole; PSC, posaconazole; ISA, isavuconazole; CAS, caspofungin; MCF, micafungin.

Susceptibility testing resulted in the following minimum inhibitory concentrations (MICs)/minimum effective concentrations (MECs) range for the tested antifungal agents: AMB (0.5–4 mg/L), voriconazole (VRC; 0.5–4 mg/L), posaconazole (PSC; 0.06–0.5 mg/L), isavuconazole (ISA; 0.25–4 mg/L), caspofungin (CAS; 0.125–0.5 mg/L), and micafungin (MCF; 0.03–0.06 mg/L) (Table 1). Comparison of the MIC/MEC ranges for each antifungal across different series revealed that the *Ambigui* series exhibited the lowest MIC range for all tested antifungals, while no significant differences were observed between series *Terrei* and *Nivei* (Table 1). Based on the latest epidemiological cut-off value (ECOFF) outlined by European Committee on Antimicrobial Susceptibility Testing (EUCAST), all strains were classified as wild type for AMB, and all were also categorized as wild type for VRC, except *A. niveus*. Notably, *A. niveus* was the only species non-wild type/resistant to all tested azoles (Fig. 1; Table 1). Due to the absence of clinical breakpoints and ECOFF for echinocandins, the susceptibility results are only shown in Table 1 and not in Fig. 1.

### Differential virulence potential of section *Terrei* in *G. mellonella*

#### Survival assays

**Fig 2:**
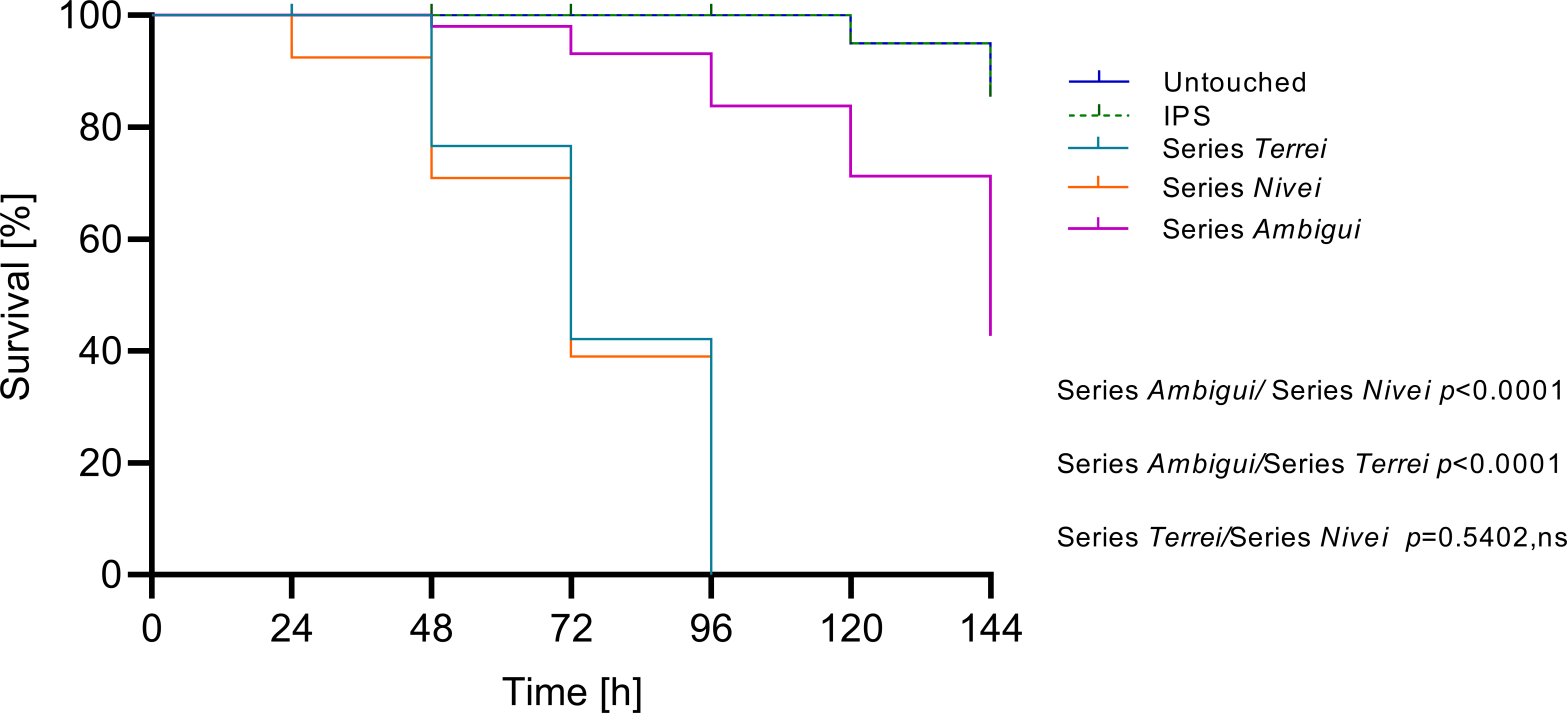
Median survival curves of series *Terrei* (included species *n* = 10), *Nivei* (included species *n* = 7), and *Ambigui* (included species *n* = 2). Control groups include untouched larvae and survival of larvae inoculated with insect physiological saline (IPS). Median survival rates were calculated from all tested species within each series across three independent experiments. *P* values for significantly different results are shown (*P* < 0.05; Mantel-Cox test); otherwise, results are marked as not significant (ns).

**Fig 3:**
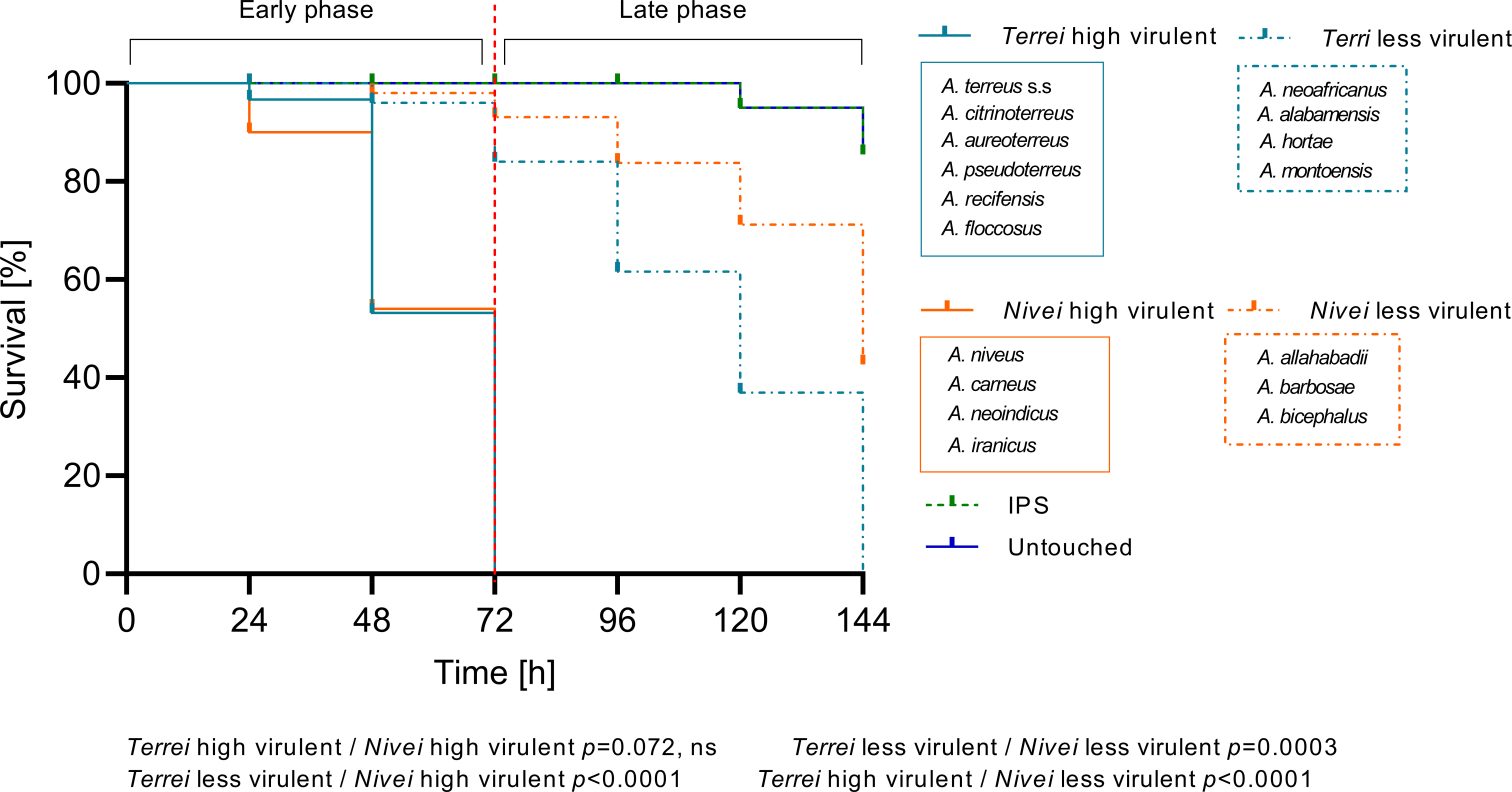
Median survival curves for series *Terrei* (included species *n* = 10) and *Nivei* (included species *n* = 7), differentiating between highly and less virulent species. Control groups included untouched larvae and those inoculated with insect physiological saline (IPS). Red vertical dotted line separates two phases of the survival curve, early (up to 72 h) and late (up to 144 h). Median survival rates were calculated from all tested species per series across three independent experiments. *P* values for significantly different results are shown (*P* < 0.05; Mantel-Cox test); otherwise, results are marked as not significant (ns).

Comparing the median survival curves across the series, series *Ambigui* showed a significant difference from *Terrei* and *Nivei* series (*P* < 0.0001), likely due to the smaller number of species in series *Ambigui*. Comparing the entire median survival curves of series *Terrei* and *Nivei*, no significant difference was observed (Fig. 2). Our detailed observation revealed that in many species, 100% mortality was reached at 72 h. Therefore, we selected 72 h post-infection as a reference time point and could see that the median survival curves of each series can be divided into two distinct phases, categorizing species into two virulence groups based on survival duration: highly virulent (short survival, up to 72 h) and less virulent (long survival, up to 144 h) (Fig. 3). The median survival rates of highly virulent species differed significantly from those of less virulent species within each series, *Terrei* and *Nivei* (Fig. 3). Both categories, highly and less virulent species in the *Terrei* series showed significant differences from the *Nivei* series as a whole (*P* = 0.0001). Although there was a significant difference between less virulent species in series *Terrei* and less virulent species in series *Nivei* (*P* = 0.0003), no significant difference was observed between highly virulent species in series *Terrei* and *Nivei*.

**Fig 4:**
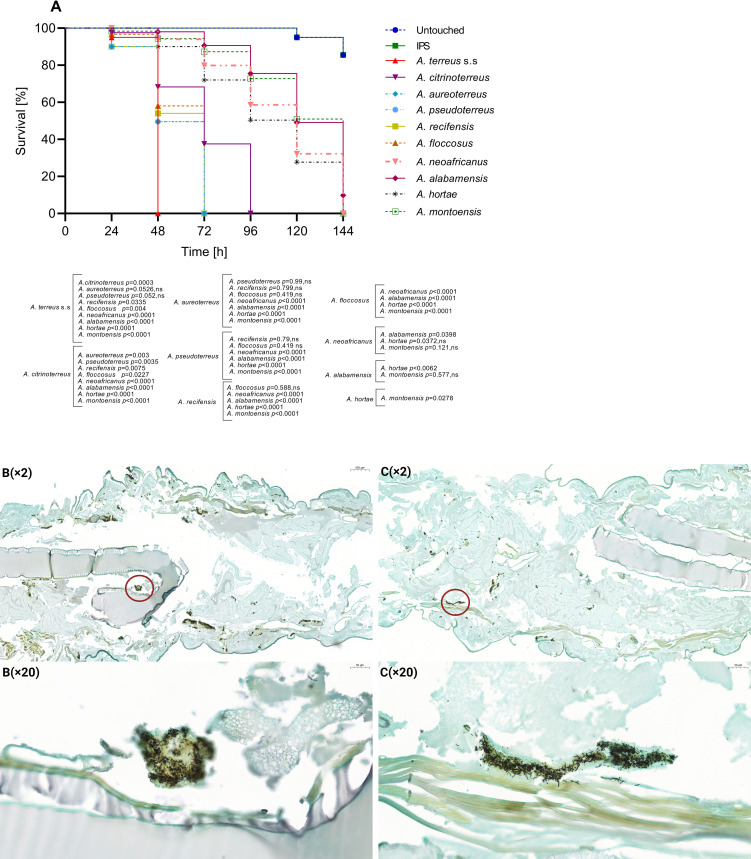
Survival curves of *Galleria mellonella* infected with *Aspergillus* species belonging to series *Terrei* (10^7^ spores/larvae). Curves represent the average of three independent experiments. *P* values for significantly different results are shown (*P* < 0.05; Mantel-Cox test); otherwise, results are marked as not significant (ns). Control groups include untouched larvae and survival of larvae inoculated with insect physiological saline (IPS) (**A**). Histopathology of *G. mellonella* larvae infected with *A. terreus* s.s., as the most virulent representative, 24 h post-infection (**B**) and *A. alabamensis,* as the less virulent representative, 72 h post-infection (**C**), circles indicating hyphal formation. Larvae fixed in formaldehyde and embedded in paraffin were longitudinally cut (5 µm thickness) and stained with Grocott (methenamine) silver (GMS). Photomicrographs were taken by 3DHISTECH’s CaseViewer software, at different magnifications ×2 and ×20.

**Fig 5:**
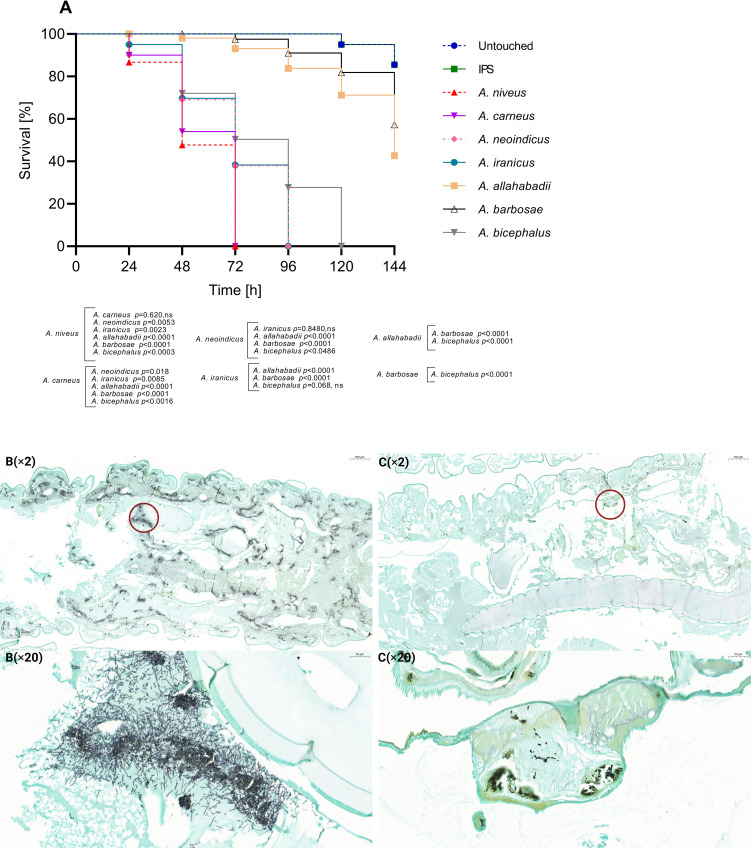
Survival curves of *Galleria mellonella* infected with *Aspergillus* species belonging to series *Nivei* (10^7^ spores/larvae). Curves represent the average of three independent experiments. *P* values for significantly different results are shown (*P* < 0.05; Mantel-Cox test); otherwise, results are marked as not significant (ns). Control groups include untouched larvae and survival of larvae inoculated with insect physiological saline (IPS) (**A**). Histopathology of *G. mellonella* larvae infected with *A. niveus*, as the most virulent representative, 24 h post-infection (**B**) and *A. barbosae,* as the less virulent representative, 72 h post-infection (**C**), circles indicating hyphal formation. Larvae fixed in formaldehyde and embedded in paraffin were longitudinally cut (5 µm thickness) and stained with Grocott (methenamine) silver (GMS). Photomicrographs were taken by 3DHISTECH’s CaseViewer software, at different magnifications ×2 and ×20.

**Fig 6:**
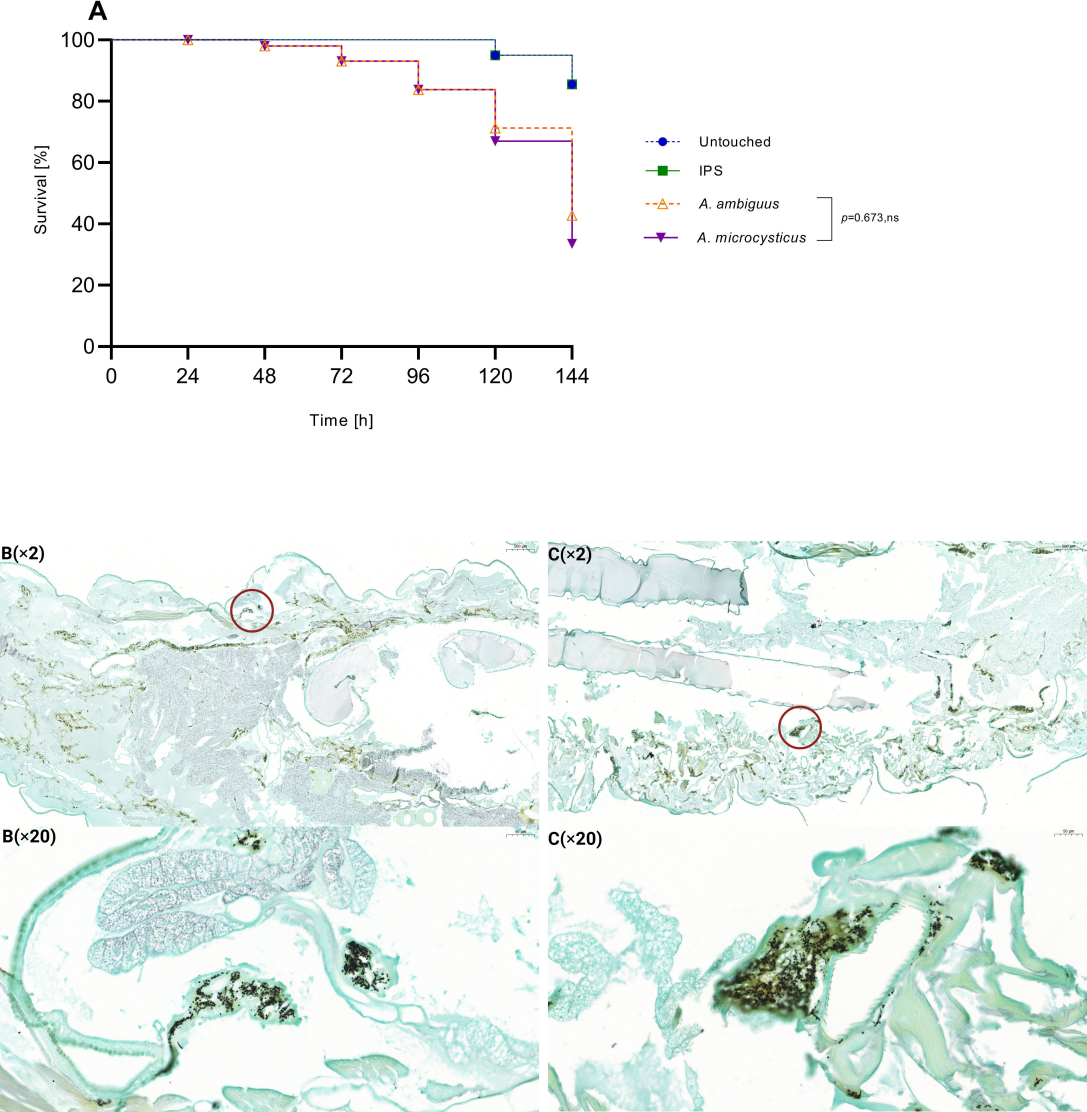
Survival curves of *Galleria mellonella* infected with *Aspergillus* species belonging to series *Ambigui* (10^7^ spores/larvae). Curves represent the average of three independent experiments. *P* values for significantly different results are shown (*P* < 0.05; Mantel-Cox test); otherwise, results are marked as not significant (ns). Control groups include untouched larvae and survival of larvae inoculated with insect physiological saline (IPS) (**A**). Histopathology of *G. mellonella* larvae infected with both existing species in series *Ambigui*; *A. ambiguus*, 72 h post-infection (**B**) and *A. microcycticus*, 72 h post-infection (**C**), circles indicating hyphal formation. Larvae fixed in formaldehyde and embedded in paraffin were longitudinally cut (5 µm thickness) and stained with Grocott (methenamine) silver (GMS). Photomicrographs were taken by 3DHISTECH’s CaseViewer software, at different magnifications ×2 and ×20.

Based on the average survival of the tested species within each series, *A. terreus*, *A. aureoterreus*, and *A. pseudoterreus* in series *Terrei* were significantly more virulent compared to other species (Fig. 4A). Conversely, *A. alabamensis*, *A. neoafricanus*, *A. hortae*, and *A. montoensis* exhibited significantly lower virulence potential compared to the more virulent species (Fig. 4A) (*P* < 0.0001). In series *Nivei*, *A. niveus* and *A. carneus* had an average survival time of 72 h, resulting in 100% mortality and indicating the highest virulence compared to other species (Fig. 5A). In contrast, *A. barbosae* and *A. allahabadii* had respective mortality rates of 39.1% and 57.3% with an average survival time of 144 h, reflecting reduced virulence compared to *A. niveus* and *A. carneus* (Fig. 5A) (*P* < 0.0001). Both species in series *Ambiguui*, *A. ambiguus*, and *A. microcyticus* exhibited reduced virulence compared to species from the other series, with survival times of 144 h and mortality rates of 57.3% and 66.5%, respectively, and no significant difference in virulence between them (Fig. 6A).

#### Histopathology

Grocott methenamine silver (GMS) staining was employed to examine the fungal distribution and filamentation parallel to the survival assay. Photomicrographs of representative species from each series, one exhibiting the highest virulence and one showing the lowest virulence potential, as determined by the survival assay, are provided for comparison. Different time points for larval sacrification were chosen for each species based on their respective survival rates, ensuring larvae had reached an advanced stage of infection while still maintaining the required tissue integrity for histological examination. In the infection due to the series *Terrei* species, notable dissemination was observed in *A. terreus*, a highly virulent representative, during the initial stages, 24 h after inoculation (Fig. 4B). In comparison, *A. alabamensis*, a less virulent representative, exhibited reduced tissue distribution during the intermediate stages after inoculation, 72 h (Fig. 4C). Both species exhibited a concentrated pattern of filamentation spots (Fig. 4B and C). In the context of the series *Nivei*, *A. niveus*, one of the most virulent representatives, demonstrated rapid and aggressive infection progression (Fig. 5B). As early as 24 h post-infection, hyphal growth was evident in the larvae. The distribution of fungal elements encompassed the entire larvae, leading to tissue invasion across nearly all areas (Fig. 5B). In contrast, *A. barbosae*, one of the least virulent representatives in the series *Nivei*, exhibited a minimal degree of filamentation and only a few concentrated conidia or short hyphae spots in the tissues after 72 h post-inoculation (Fig. 5C). Both species in the series *Ambiguui*, *A. ambiguus* and *A. microcycticus*, showed low tissue invasion in surviving larvae up to 72 h post-infection (Fig. 6B and C). The species displayed a non-aggressive behavior; however, fungal elements could reach the entire tissue, forming various colonization spots primarily in conidial form and to a lesser extent in filamentous form (Fig. 6B and C).

## DISCUSSION

The *Aspergillus* genus comprises numerous species, some of which have pathogenic potential and are associated with invasive fungal infections (IFIs) (19, 20). Historically, human aspergillosis mostly involved a limited number of *Aspergillus* species, with *A. fumigatus* being the most common, pursued by less common non-*fumigatus* species, such as those in section *Terrei*, namely, *A. terreus* (3, 21). *A. terreus* is associated with a higher rate of dissemination compared to non-*terreus Aspergillus* species and also poses treatment challenges due to its reduced susceptibility to AmB (8, 22–24). The emergence of cryptic *Aspergillus* species, which cannot be distinguished based solely on phenotypic traits, has become increasingly apparent in clinical settings (18, 25). Multicenter investigations have indicated that cryptic *Aspergillus* species were implicated in approximately 10–30% of invasive cases (15, 26). This trend is attributed to the growing immunocompromised population and the application of advanced molecular techniques in taxonomy and diagnostics (27, 28). Importantly, some studies have shown that cryptic species of *Aspergillus* display diminished susceptibility to conventional antifungals, potentially altering treatment outcomes (27–30). This underscores the importance of accurately identifying and characterizing cryptic species. As these species could be associated with invasive disease, making them significant in both epidemiological and clinical contexts (31–33). Understanding the characteristics of cryptic species is crucial for conducting clinical management strategies, especially when confronting uncommon species.

Previously, it has been indicated that the most commonly isolated species within section *Terrei*, both clinically and environmentally, were members of the series *Terrei*, such as *A. terreus* s.s., *A. citrinoterreus*, and *A. hortae* (34–36). The last two of these can be considered cryptic species that could potentially be overlooked in less experienced centers. Variations in species frequencies in clinical samples may reflect their higher abundance in the environment, leading to heightened exposure among vulnerable patients. However, the ecological niches and epidemiology of species within section *Terrei* require further in-depth investigation (36, 37). As IFIs become more prevalent, there is an increasing need for accurate identification of less common *Aspergillus* species, coupled with a thorough understanding of their epidemiological cutoff values.

The growing prevalence of antifungal resistance, combined with the limited availability of therapeutic antifungal agents, renders the treatment of IFIs challenging (10, 38, 39). Selecting an antifungal agent for therapy depends on the fungal pathogen’s susceptibility to the antimycotic agent, emphasizing the importance of accurate species identification and susceptibility testing to guide the appropriate therapy (40). Assessing the antifungal susceptibility profiles of each series separately revealed that series *Ambigui* exhibited the narrowest MIC range for all antifungals compared to the other two series (Table 1), which could be probably mainly attributed to the lower number of species (two species). Furthermore, although there were no notable differences in the susceptibility profiles of series *Terrei* and *Nivei*, series *Nivei* displayed one-step higher MIC range for azoles compared to series *Terrei* and *Ambigui*. Particularly, *A. niveus* showed the highest VRC and ISA MIC values, followed by *A. iranicus* with a high ISA MIC (Table 1). This observation aligns with previous studies demonstrating the association of *Aspergillus* genus cryptic species with reduced azole susceptibility (32, 41). The clinical significance of these findings is further supported by the study revealing *A. niveus* as a causative agent of pulmonary aspergillosis (42).

*G. mellonella* larvae have been extensively utilized as an alternative model to assess the virulence of fungal pathogens, including various *Aspergillus* species (43–45), with the pros and cons of this model extensively discussed elsewhere (46–49). In the present investigation, the pathogenicity of species within each series was determined using a larval infection assay by comparing survival durations. Besides, histological studies were conducted to characterize the invasive nature of the infection (50). Overall, the results of the larval infection assay were consistent with histological findings, revealing tissue lesions, fungal distribution, and filamentation. Our findings are consistent with prior research emphasizing the importance of characterizing less common species and those belonging to diverse taxonomical clades (51–54).

When considering the overall median survival data for each series, encompassing all tested species per each series, series *Ambigui* was found to differ significantly from both the *Terrei* and *Nivei* series (Fig. 2). At the 72 h midpoint of the survival curve, distinct trends were recognized among species of series *Terrei* and *Nivei*, distinguishing between two survival patterns: high virulent species with survival up to 72 h and less virulent species with survival extending up to 144 h (Fig. 3). Comparative analysis between the *Terrei* and *Nivei* series revealed a significant difference in the survival rates of less virulent species, while no significant difference was observed between the highly virulent species in the two series (Fig. 3). *A. terreus* s.s., *A. aureoterreus*, and *A. pseudoterreus* were identified as the most virulent members of the series *Terrei* (Fig. 4A). *A. aureoterreus* and *A. pseudoterreus*, typically classified as cryptic species of *A. terreus*, demonstrated notable virulence, as evidenced by their comparable survival rates to *A. terreus* s.s (Fig. 4A), with no noticeable differences observed in the histological examination compared to *A. terreus* s.s. (data not shown).

Within the series *Nivei*, phylogenetically close species *A. niveus* and *A. carneus* displayed high virulence, resulting in a 100% mortality rate at 72 h (Fig. 5A). However, upon histological examination, *A. niveus* exhibited an unusual distribution of hyphae (Fig. 5B), extending beyond necrosis spots to permeate throughout the entire larvae, indicating heightened invasiveness and emphasizing the species' potential for dissemination. The elevated azole MIC profile of *A. niveus*, coupled with its survival rate and histological characterization, emphasizes its high pathogenic potential, which is in line with the previous study (42), despite its infrequent discussion within section *Terrei*. Series *Ambigui* displayed the lowest virulence, with prolonged survival rates (Fig. 6A) and reduced invasiveness in histological observations (Fig. 6B and C). Although fungal elements were distributed throughout the entire larvae, less filamentation in the spots was observed, highlighting the importance of the transition from conidial to mycelial forms in pathogenicity (55).

The high virulence potential of some cryptic species revealed in this study, for example, virulence of *A. aureoterreus* and *A. pseudoterreus* comparable to *A. terreus* s.s., underscores the significance of cryptic species awareness for preparedness in case of emergence. Lackner et al. (46) examined 73 isolates from six species within the *Terrei* series and found that cryptic species exhibited similar virulence in *G. mellonella*, with differences attributed to strain rather than species. While this study likely represents the most comprehensive analysis of pathogenicity within *Aspergillus* section *Terrei*, considering both survival and histological aspects, the number of tested strains per species is low and mostly involves only ex-type strain of every species. Inclusion of more isolates per species with diverse phenotypes would enable to capture the potential heterogeneity within species and to study virulence from the ecological perspective (e.g., potential differences between environmental and clinical isolates). Furthermore, the primary objective of the histological approach in this study was qualitative, necessitating quantitative investigations to validate the findings and analyze the immune response, as well as fungal burden *in vivo*.

### Conclusion

In summary, this study provides new insights into the virulence characteristics of *Aspergillus* section *Terrei*, revealing significant differences among species from distinct clades. Some cryptic species exhibit high virulence and reduced susceptibility to antifungals, underscoring the importance of differentiating cryptic species from well-known species from a clinical perspective. Further studies incorporating an extended number of isolates per species, both *in vitro* and *in vivo*, are necessary to unravel the pathogenicity mechanisms of *Aspergillus* section *Terrei*.

## MATERIALS AND METHODS

### Fungal strains and antifungal susceptibility testing

Nineteen accessible species, mostly ex-type strains, from the currently accepted species in section *Terrei* were analyzed (the details of strains in Table 1). Strains were provided by the CBS biobank housed at the Westerdijk Fungal Biodiversity Institute, Utrecht, the Netherlands. Strains were identified as previously described (9, 20). Cryopreserved strains maintained in 10% glycerol at −80°C were cultivated on malt extract agar (Carl Roth, Karlsruhe, Germany) at 37°C for up to 5 days. Subsequently, spores were harvested utilizing a spore buffer composed of 0.9% NaCl and 0.01% Tween 20 (Sigma-P1379). Antifungal susceptibility testing was performed following the broth microdilution methodology established by EUCAST Def. 9.3.2 (56). The used antifungals were AMB (range 0.03–16 mg/L; Sigma-Aldrich, A2411), PSC (0.016–8 mg/L; Sigma-Aldrich, SML 2287), VRC (0.03–16 mg/L; Sigma-Aldrich, PZ0005), ISA (0.03–16 mg/L; Sigma-Aldrich, SML 2357), CAS (0.03–16 mg/L; Sigma-Aldrich, SML 0425), and MCF (0.03–16 mg/L; Sigma-Aldrich, SML 2268). The MIC, the concentration at which no hyphal growth was detected, was assessed for AMB, PSC, VRC, and ISA, and for echinocandins, the MEC, which markedly altered hyphal growth with blunted colonies, was assessed. A final reading of the MIC/MEC results was performed after 48 h. Susceptibility testing was conducted in duplicate, and the highest value was reported.

### Phylogenetic analyses

Phylogenetic analysis was conducted following previously established methods (57). Accession numbers to sequences derived from the ex-type strains of respective species were retrieved in the curated lists of accepted *Aspergillus* species (9) and downloaded from the GenBank database. Briefly, the sequences were aligned in MAFFT v.7 (58) using the G-INS-I strategy. The best-fitting substitution models were selected using the Bayesian information criterion: the K80+G model was chosen for the *BenA* alignment; TrNef+G for the *CaM* alignment; and TrNef+G for the *RPB2* alignment. A maximum likelihood (ML) tree was constructed in IQ-TREE v.2.1.2 (59). Branch support was determined by 1,000 standard bootstrap replicates. The graphical output was created in iTOL v.6.5.6 (60) with color strips representing the resistance/susceptibility or WT/non-WT status of species based on available clinical breakpoints/ECOFF.

### *G. mellonella* infection assay

Healthy sixth instar larvae of *G. mellonella*, weighing between 0.3 and 0.4 g (SAGIP, Italy), were selected and maintained at 15°C in the dark conditions prior to the experiment. Infection assays in *G. mellonella* were performed following previous protocols (44, 46). Briefly, fungal strains were grown on malt extract agar (Carl Roth, Karlsruhe, Germany) for up to 5 days at 37°C. Spore solutions were filtered through 40 µm (PluriSelect Life Science, Germany) and 5 µm (Sysmex, Germany) cell strainer, respectively, and washed three times with spore buffer to eliminate hyphae and conidiophores. Following spore counting with a hemocytometer, the suspension was adjusted to a concentration of 5 × 10^8^/mL in insect physiological saline (IPS; 150 mM NaCl, 5 mM KCl, 10 mM EDTA, and 30 mM sodium citrate in 0.1 M Tris-HCl [pH 6.9]). Two control groups were included: untouched larvae and larvae injected with 20 µL of sterile IPS. The survival rate was monitored every 24 h for up to 144 h. Larvae were incubated at 37°C in the dark, with 20 larvae injected per test group for each run. Experiments were conducted in triplicates, and data from all experiments were combined to calculate the average and median survival rates at 24 h intervals over the 144 h observation period. Average or mean values of 0.5 or above were rounded up to the next higher number.

### Histology of *G. mellonella*

Initially, the optimal time point for sample collection was determined based on the species-specific survival curve. For highly virulent species, sample collection was performed at the earliest feasible time point, 24 h post-inoculation, while for the least virulent species, collection occurred at the intermediate time point of 72 h, for both categories ensuring larvae were not yet in a softened condition. Subsequently, infected larvae (three larvae per each test group), along with control larvae injected with sterile IPS incubated at 37°C, were injected with formalin (100 µL of 10% buffered formalin in phosphate-buffered saline) and collectively preserved intact in formalin for a duration of 10 days. Then, the larvae embedded in paraffin were longitudinally sectioned at 5 µm thickness and subjected to GMS to facilitate the detection of fungal elements (46). The scanned histological samples were analyzed by a digital microscopy application, 3DHISTECH’s CaseViewer and photomicrographs were taken at different magnifications.

### Statistical analysis

Survival rates of *G. mellonella* were determined using Kaplan-Meier survival curves and analyzed with the log-rank (Mantel-Cox) method, utilizing GraphPad Prism version 9.0.0 for Windows (GraphPad Software, San Diego, CA, USA). *P* values < 0.05 were considered statistically significant. BioRender was utilized to design the multi-panel figures.

## Data Availability

All data pertinent to the study are included in the article.
